# Posterior pituitary tumors: review of histogenetic continuum, molecular drivers and targetable therapies

**DOI:** 10.1007/s11102-026-01786-3

**Published:** 2026-11-13

**Authors:** Elisa Lamback, Anna Clara Castro Araujo, Mônica R. Gadelha

**Affiliations:** 1 Neuropathology and Molecular Genetics Laboratory, Instituto Estadual do Cérebro Paulo Niemeyer, Secretaria Estadual de Saúde, Rio de Janeiro, Brazil; 2 Neuroendocrine Unit, Instituto Estadual do Cérebro Paulo Niemeyer, Secretaria Estadual de Saúde, Rio de Janeiro, Brazil; 3 Neuroendocrinology Research Center, Endocrinology Section, Medical School and Hospital Universitário Clementino Fraga Filho, Universidade Federal do Rio de Janeiro, Rio de Janeiro, Brazil; 4 Neuropathology and Molecular Genetics Laboratory, Instituto Estadual do Cérebro Paulo Niemeyer, Rua do Rezende, 156, Centro, Rio de Janeiro, 20231-092 Brazil

**Keywords:** Posterior pituitary tumors, Spindle cell oncocytoma, Granular cell tumor, Pituicytoma, Ependymal pituicytoma, TTF-1

## Abstract

**Background:**

Posterior pituitary tumors (PPTs) are rare sellar neoplasms arising from pituicytes. Historically classified as distinct benign entities, including pituicytoma (or classical pituicytoma), spindle cell oncocytoma (SCO), granular cell tumor (GCT) of the sellar region, and ependymal pituicytoma, recent World Health Organization updates have unified them within a single pathological spectrum.

**Methods:**

This review comprehensively synthesizes the evidence supporting the spectrum concept, integrating clinical, histopathological, genomic, epigenomic, and tumor immune microenvironmental findings of pituicytomas, SCOs and GCTs. As ependymal pituicytomas are extremely rare, we synthetize their findings separately at the end of the review.

**Results:**

Clinically, PPTs may mimic non-functioning pituitary adenomas or present as suprasellar masses, and can cause neurological and endocrine deficits due to mass effect. Surgery remains the treatment of choice, with radiotherapy considered for selected patients. Recent molecular studies have provided important insights into their biology. Chromosomal copy number variations (CNV) distinguished PPTs into three major prognostic subtypes: CNV-negative pituicytomas and SCOs associated with more favorable outcomes than the CNV-positive subtypes, and a third group composed of GCTs that have favorable outcomes. Additionally, PPTs exhibit substantial driver diversity: pituicytomas and SCOs are enriched for pathogenic variants in the mitogen-activated protein kinase (MAPK) and phosphoinositide 3-kinase (PI3K) signaling pathways associated with downstream phosphorylated extracellular signal-regulated kinase (pERK) activation, whereas GCTs are characterized by relative genomic stability. Moreover, SCOs display an immune-active microenvironment, contrasting with the highly immunosuppressive profile observed in pituicytomas.

**Conclusion:**

These genomic and immune discoveries provide a rational for targeted therapy for refractory PPTs.

## Introduction

Neoplasms of the sellar region comprise a wide variety of tumor types, including the very common pituitary adenomas (PAs), craniopharyngiomas, and less common posterior pituitary tumors (PPTs) [1]. PPTs are exceedingly rare, accounting for less than 1% of surgically resected sellar masses [2].

PPTs are rare non-neuroendocrine and low-grade neoplasms, that were initially recognized as distinct tumors with no clear histogenetic relation, each with its own theories regarding cellular origin [3–5]. Pituicytoma was conceptualized as a low-grade astrocytic tumor of neurohypophyseal glial cells [6]. Granular cell tumor (GCT) of the sellar region (historically termed choristoma or Abrikossoff tumor) was classified as an indolent, lysosome-rich neoplasm postulated to arise from Schwann cells or neuroepithelial elements [3]. Spindle cell oncocytoma (SCO) was originally described by Roncaroli et al. in 2002 as an oncocytic neoplasm of the anterior pituitary presumably arising from folliculostellate cells [4]. Sellar ependymoma (now termed ependymal pituicytoma) was regarded as an ectopic ependymoma mimicking its spinal and intraventricular counterparts [7, 8].

A major conceptual shift came with the study by Lee et al. published in 2009, that demonstrated that pituicytomas, SCOs, and GCTs share a common, diffuse nuclear expression of the homeobox transcription factor thyroid transcription factor-1 (TTF-1), also present in adult human pituicytes, the glial cell of the neurohypophysis and infundibulum [9]. This discovery led to the hypothesis that these tumors were not unrelated entities but rather represented morphological and functional variants of a single pituicytic lineage. This histogenetic spectrum hypothesis was recognized in the fourth and fifth editions of the World Health Organization (WHO) Classification of Central Nervous System Tumors (CNS4 and CNS5) [10]. Sellar ependymoma was not included in CNS5 because of its rarity [10]. However, the subsequent edition of the WHO classification of Endocrine Tumors (ENDO5) included sellar ependymoma as part of PPT family, due to shared expression of TTF-1 [11]. Therefore, PPTs are now grouped as neoplasms derived from pituicytes from the posterior pituitary and infundibulum, comprising four histopathologically defined subtypes: pituicytoma (also known as classical pituicytoma), granular cell tumor of the sellar region (or granular cell pituicytoma), spindle cell oncocytoma (or oncocytic pituicytoma), and ependymal pituicytoma (previously known as sellar ependymoma) [12, 13].

Due to their rarity, the precise incidence of PPT remains unknown [10]. In an autopsy study, GCTs were found in 13 out of 150 autopsies with an incidence of 8.8%, usually small (0.55 mm) [14]. No pituicytoma or SCO were encountered in this study. In other autopsy studies, GCTs were the most frequent PPTs found in unselected autopsies, present in about 5–6% of patients [14]. A systematic review conducted in 2019 identified 259 patients of PPTs described in the literature, including 135 pituicytomas, 69 GCTs, 47 SCOs and eight ependymal pituicytomas [15]. Tumors with mixed histology have also been described in this systematic review in 2.6% of cases [15]. More than 70% of reported cases have been published within the past decade, reflecting the relatively recent recognition of these rare neoplasms [2].

As ependymal pituicytoma are extremely rare, we will focus on pituicytoma, GCT and SCO describing their clinical, radiological, histomolecular and immune findings together, and at the end of the review, summarize findings on ependymal pituicytoma.

## Clinical presentation

The median age at presentation varies slightly between subtypes, with GCTs presenting earlier (median age of 42.5 years, range 27–77) compared to pituicytomas and SCOs, which occur predominantly at a median age of 58 (range 31–76) [16–20]. SCO and pituicytomas appear to affect both genders equally [21, 22], while GCT show a slight female predilection (57% of 88 reported cases) [23].

Due to their slow growth, PPTs are often incidentally diagnosed or present with non-specific symptoms resulting from progressive compression of adjacent structures [17, 21, 24–26]. Exceptionally rare are functioning PPT with symptoms of hypercortisolism or acromegaly [15, 27, 28].

The clinical presentation of PPTs closely mimics that of non-functioning PAs [29]. The most frequent symptoms are visual field deficits (present in 50–60% of the patients), including bitemporal hemianopia, and chronic headache (present in 25–40% of the patients) [12, 13]. Hypopituitarism is common (22–40% of patients), resulting from direct compression of the anterior pituitary gland or the portal vessels within the pituitary stalk [13]. Central hypogonadism is the commonest pituitary deficiency, but central adrenal insufficiency, hypothyroidism, and growth hormone deficiency can also be observed [13, 20, 30].

A striking clinical hallmark of PPTs is the low incidence of preoperative arginine vasopressin deficiency (AVP-D), despite the tumors originating directly within the neurohypophysis or the infundibular stalk [29, 31]. In large systematic reviews and patient series, preoperative AVP-D is reported in less than 5% of patients [2]. Arginine vasopressin (AVP) is synthesized in the cell bodies of magnocellular neurons located in the supraoptic and paraventricular nuclei of the hypothalamus. The posterior pituitary consists merely of the terminal axons of these neurons. Progressive tumor growth in the sellar region causes gradual compression of these axonal terminals, while the proximal axonal segments and hypothalamic cell bodies remain functionally intact. AVP is released *en-passant* from axonal terminals within the internal zone of the median eminence, thereby preserving vasopressin secretion and preventing hormonal deficiency [32]. However, AVP-D is a frequent postoperative complication, occurring in up to 30% of patients following transsphenoidal or transcranial surgical resection due to intraoperative traction on the infundibulum or surgical disruption of the proximal pituitary stalk [29, 30].

## Neuroimaging

PPTs are usually misdiagnosed as more common sellar lesions, particularly PAs [20, 33, 34]. Sellar magnetic resonance imaging (MRI) is the modality of choice for evaluating the sellar region and may help distinguish PPTs from other tumors [35].

**Fig. 1:**
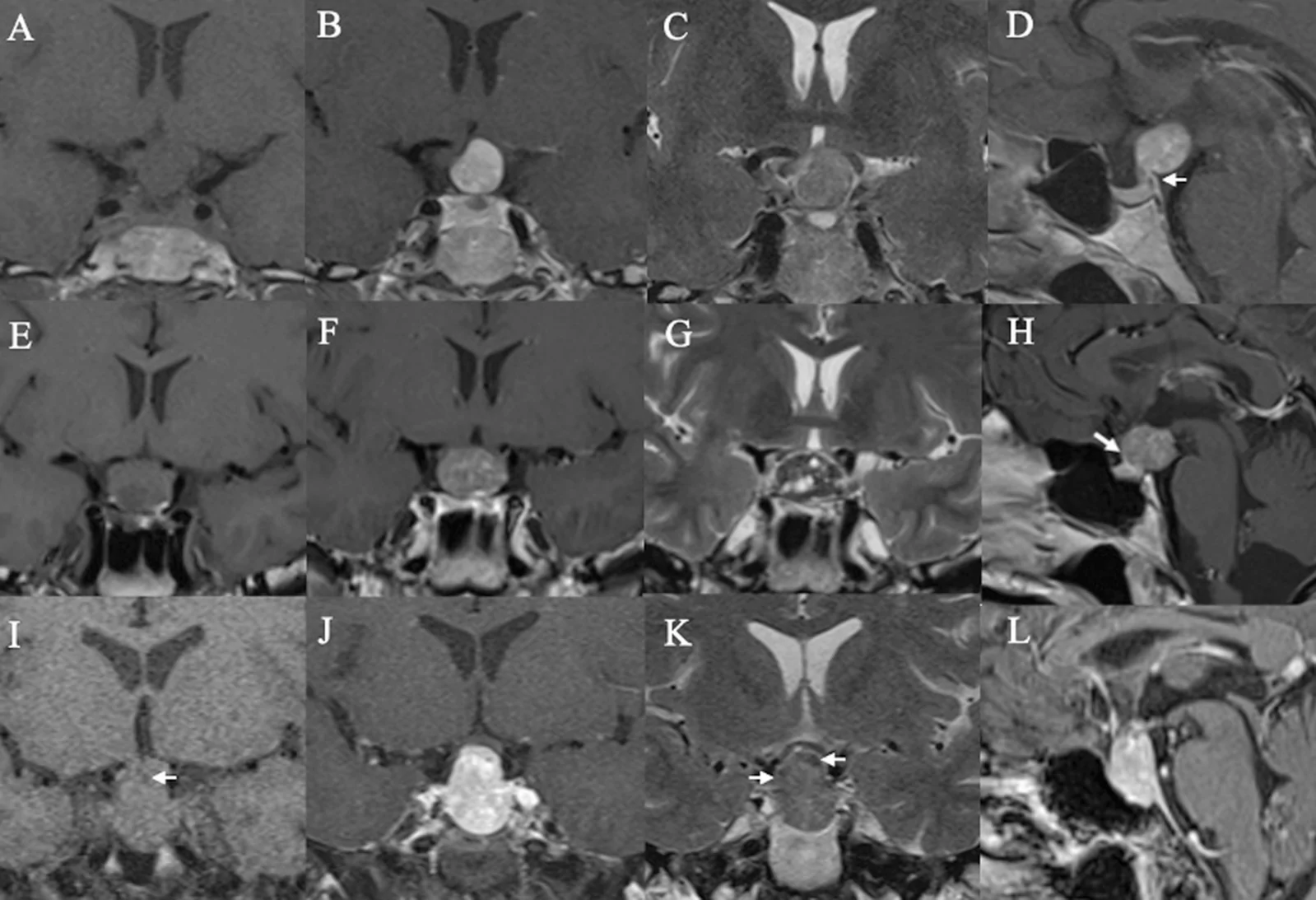
Magnetic resonance imaging of posterior pituitary tumors. Pituicytoma: Coronal T1W (**A**), coronal T1W post-contrast (**B**), coronal T2W (**C**), and sagittal T1W post-contrast (**D**) images demonstrating a suprasellar and posterior lesion, isointense on T1W and T2W, with homogeneous contrast enhancement, located at the distal end of the pituitary stalk (arrow in D). Granular cell tumor: Coronal T1W (**E**), coronal T1W post-contrast (**F**), coronal T2W (**G**), and sagittal T1W post-contrast (**H**) images demonstrating a suprasellar lesion, predominantly solid in composition, isointense on T1W, with small foci of cystic degeneration and heterogeneous contrast enhancement, that grows posteriorly and is indissociable from the pituitary stalk (arrow in H), with an inverted drop shape. Spindle cell oncocytoma: Coronal T1W (I), coronal T1W post-contrast (**J**), coronal T2W (**K**), and sagittal T1W post-contrast (**L**) images showing a predominantly sellar lesion with suprasellar extension, isointense on T1W and T2W, with hypointense foci (Hasiloglu’s sign - arrows in I and K), showing intense contrast enhancement (J, L)

PPTs typically present as solid, well-demarcated lesions located in the sellar and suprasellar region – Table 1; Fig. 1 [36]. Pituicytomas are mostly round or oval lesions, centered in the suprasellar region, isointense to cerebral gray matter on T1-weighted imaging (T1W) and iso- to slightly hyperintense on T2W, with homogeneous contrast enhancement [13]. They can have avid contrast-enhancement as described by Feng et al. [37] or not [20].

**Table 1:** Summary of radiological and histological findings in posterior pituitary tumors

	Pituicytoma	Granular cell tumor	Spindle cell oncocytoma	Ependymal pituicytoma
Approximate number of tumors	150	80	50	<20
Magnetic resonance imaging	• **Round or oval**,** centered in the suprasellar region** • **Homogeneous contrast enhancement** • **Isointense on T1W** • **Iso- to slightly hyperintense on T2W**	• **Suprasellar centered on the pituitary stalk or infundibulum** • **‘Star-like crack sign’** • **Inverted drop shape** • **Heterogeneous contrast enhancement** • **Iso- to markedly hypointense on T2W**	• **Large intra-sellar lesion with suprasellar extension** • **Hasiloglu’s sign** • **Avid contrast enhancement** • **Isointense on T1W and T2W**	• **Solid or cystic-solid** • **Well-defined tumor margins upon contrast enhancement** • **Hypointense on T1W and hyperintense on T2W**
Morphology	Elongated spindle-shaped cells in fascicles or bundles	Large polygonal to ovoid cells with coarse granules PAS-positive	**Spindle-to-epithelioid cells with abundant mitochondria and oncocytic change**	**Spindle cell architecture with ependymal rosettes and perivascular pseudorosettes**
Staining	• Positive: TTF-1 (diffuse), S100, vimentin • Variable: GFAP	• Positive: TTF-1 (diffuse), CD68, alpha1-antitrypsin • Variable: S100, vimentin, GFAP	• Positive: TTF-1 (diffuse), S100, vimentin, AMA • Variable GFAP, EMA	• Positive: EMA (dot-like/apical) • Variable: TTF-1 (diffuse to focal), S100, vimentin, GFAP

*AMA* anti-mitochondrial antibody, *EMA* Epithelial membrane antigen; *GFAP* Glial fibrillary acidic protein; *NSE* neuron-specific enolase; *PAS* periodic acid-Schiff

In contrast, GCTs and SCOs often present with heterogeneous contrast enhancement [38] GCT usually appears as a solid suprasellar mass centered at the base of the pituitary stalk, that grows upward and can compress the optic chiasm – Table 1; Fig. 1 [39]. On T2W, GCTs typically manifest as iso- to markedly hypointense lesions, which correlates pathologically with the dense packing of proteinaceous lysosomes and cytoplasmic granules [40]. Large GCT masses frequently exhibit a characteristic radiological pattern termed the ‘star-like crack sign’ [40]. This sign consists of linear, low-signal structures on T1W and high-signal structures on T2W that radiate outward from a central point, resembling a crack or a star [40]. Pathologically, the star-like crack is hypothesized to result from an unbalanced, heterogeneous distribution of mucoproteins within the tumor matrix [40]. Notably, this crack-like structure shows marked contrast enhancement after gadolinium administration due to capillary perfusion within the proteinaceous clefts [2, 20, 40]. In our experience, GCT present typically as a suprasellar lesion with an inverted drop shape [20]. The presence of a suprasellar mass located in the posterior pituitary/infundibulum, closely related to the pituitary stalk, and predominantly solid in composition, that is hypointense on T2W with a central star-like crack or with an inverted drop shape is highly indicative of GCT.

With respect to SCOs, they can appear as a large intra-sellar lesion with suprasellar extension mimicking PAs [20], purely sellar or purely suprasellar lesions [2, 26], or uncommonly as an exclusively cystic lesion [2] – Table 1; Fig. 1. SCOs are isointense on T1W and T2W, and have an intense contrast-enhancement described as avid or hypervascular, indicating a well-developed capillary network [15, 26, 33, 41]. Another distinctive radiological marker of SCO is the Hasiloglu’s sign that corresponds to multiple, millimetric hypointense foci and linear signal-void areas on T1W and T2W [42]. Pathologically, the hypointense millimetric foci correspond to extensive intratumoral hemosiderin deposits resulting from micro-hemorrhages, while the linear signal voids represent fast-flowing, tortuous blood vessels [42]. Exceptionally, SCOs may present as hemorrhagic masses associated with massive bleeding [33, 43–45]. Recognizing Hasiloglu’s sign preoperatively is highly valuable, as it alerts the surgical team to the high vascularity and bleeding potential of SCOs.

Distinguishing PPTs from PAs is important because of differences in surgical planning and perioperative management, as SCOs may pose additional challenges due to their high vascularity [46]. In this context, artificial intelligence and radiomics have emerged as promising tools for preoperative diagnosis. Jin et al. developed an individualized model combining radiomic features and clinical data in 110 patients with non-functioning PAs and 55 with PPTs, achieving 87.9% accuracy, 93.6% sensitivity, and 76.4% specificity, significantly outperforming radiological features alone [46]. Additionally, Liu et al. developed a radiomics-based machine learning model to distinguish PPTs from PAs and craniopharyngiomas [47]. Using 1,510 radiomic features from T1W and contrast-enhanced MRI in 42 PPTs and 132 non-PPT tumors (68 PAs and 64 craniopharyngiomas), the model achieved 78.6% accuracy, 78.8% sensitivity and 77.8% specificity [47]. These findings suggest that radiomics may provide a valuable non-invasive approach to improve preoperative diagnosis and surgical planning.

## Histopathological spectrum

The core argument surrounding the spectrum hypothesis of PPTs is rooted in their shared histogenetic lineage [13, 48]. All PPT subtypes exhibit nuclear expression of TTF-1, while being strictly negative for pituitary hormones and lineage-specific transcription factors (PIT1, TPIT, SF1), and neuronal and neuroendocrine markers, confirming their pituicytic nature [13, 48]. In Lee et al.’s original report, two monoclonal anti-TTF-1 antibodies were evaluated (clone 8G7G3/1 and clone SPT24) [9]. Both showed nuclear immunoreactivity in PPTs, however staining was weaker with clone 8G7G3/1 compared to clone SPT24. Additionally, clone 8G7G3/1 showed cytoplasmic immunoreactivity in a subset of anterior pituitary cells. Therefore, clone SPT24 was used for the subsequent analyses in this study.

Other immunohistochemical markers may aid in establishing the subtype of PPT, which is primarily based on morphological features. Although S100 and vimentin are non-specific antigens, S100 protein (marker of glial-like pituicyte) is nearly positive in every PPT, while vimentin (intermediate filament protein found in glial cells) and glial fibrillary acidic protein (GFAP; marker of glial differentiation) show variable expressions [49]. PPTs usually exhibit low mitotic activity, consistent with their low grade nature [20]. Structurally, each of the four recognized variants exhibits characteristic histopathological features – Table 1.

Pituicytoma consists of elongated spindle-shaped cells arranged in fascicles or bundles, with elongated nuclei and scant eosinophilic cytoplasm [13, 34]. Pituicytoma lack Rosenthal fibers and eosinophilic granular bodies, which helps differentiate them from other neurohypophyseal lesions, such as an astrocytoma, and lack Herring bodies which helps differentiate them from normal neurohypophysis [13]. Pituicytomas show strong reactivity for S100 and vimentin, but variable GFAP immunostaining [13, 50].

GCTs are composed of large polygonal to ovoid cells, densely packed, containing coarse, with granular eosinophilic cytoplasm that are strongly positive for periodic acid-Schiff (PAS) and highly resistant to diastase digestion, representing an accumulation of lysosomal enzymes [13, 34, 48]. The nuclei are generally small and centrally located within the cytoplasm, with usually minimal nuclear atypia [20]. Immunohistochemical staining reveals variable S100 and vimentin expression, and only occasional immunoreactivity for GFAP and epithelial membrane antigen (EMA; a heavily glycosylated transmembrane protein normally expressed on the apical surface of glandular and ductal epithelial cell). GCT are positive for CD68 (a marker of lysosomes) and alpha1-antitrypsin (marker of lysosome accumulation) [12].

SCO is characterized by interlacing bundles of spindle-to-epithelioid cells showing abundant, eosinophilic, highly granular cytoplasm packed with mitochondria-rich, oncocytic elements [13, 50]. The cells may be arranged in fascicles, whorls or sheets [20]. In our single-center experience, proliferation of vessels with thickened walls was observed in almost all cases, which can explain the intense contrast-enhancement seen in SCOs [20]. S100 and vimentin are positive, and GFAP expression can be present. EMA is variable, with focal or dotted positivity [13, 50]. Accumulation of mitochondria in SCO is confirmed by strong cytoplasmic positivity for anti-mitochondrial antibody (AMA), which differentiates it from pituicytoma (AMA-weak) and GCT (AMA-negative) [49, 51].

## Genomic and immune alterations

Pituicytomas and SCOs frequently harbor somatic driver alterations enriched in the mitogen-activated protein kinase (MAPK) and phosphoinositide 3-kinase (PI3K) signaling pathways in 6/9 tumors (66%) and 7/18 tumors (38%), respectively, not observed in any GCT [19]. High-throughput targeted sequencing has identified activating hotspot pathogenic variants in *BRAFV600E*, *FGFR1*, *HRAS*, *MAP2K2*, *CBL*, and inactivating variants in *NF1* and *PTEN*, accompanied by widespread nuclear and cytoplasmic phosphorylated extracellular signal-regulated kinase (pERK) expression – Table 2 [19, 52]. Furthermore, recent data revealed that pituicytomas are generally positive for vascular endothelial growth factor (VEGF) and somatostatin receptors (SST) type 3 and 5 [33].

**Table 2:** Integrated histomolecular classification of posterior pituitary tumors

Subtype profile	Genomic landscape	Chromosomal copy number	Prognosis (Schmid et al., 2021)
Subtype 1 (Genetically stable pituicytoma/SCO)	May harbor MAPK/PI3K pathway pathogenic variants *(FGFR1*,* HRAS*,* BRAF*,* CBL*,* NF1).*	Strictly CNV-negative (No chromosomal gains or losses).	Highly favorable course. Median follow-up 2.8 years. Only 1 recurrence observed. Low risk.
Subtype 2 (Genetically unstable pituicytoma/SCO)	May harbor MAPK/PI3K pathogenic variants and epigenetic regulators (*SMARCB1*).	CNV-positive (chromosomal imbalances present; frequent 1p loss in SCO).	Less favorable course. Median follow-up 7 years. 47% (8/17) recurred. High risk.
Subtype 3 (GCT group)	Genomically silent. Rare pathogenic variants.	Typically CNV-negative (few or no copy number alterations).	Favorable outcomes. Median follow-up 4.7 years. Only 2 recurrences observed. Low risk.

*CNV* copy number variation; *GCT *Granular cell tumor, *SCO *Spindle cell oncocytoma

In contrast to pituicytomas and SCOs, GCTs are largely genomically silent regarding somatic pathogenic variants; instead, they exhibit subtle epigenetic deregulation marked by differentially methylated regions [19]. Whole-exome sequencing of GCTs has identified rare variants in oncogenes *SETD2* and *PAX8* [53].

Regarding the tumor microenvironment, Li et al. studied the immune landscape of 16 PPTs (10 pituicytomas and six SCOs), focusing on the infiltration patterns of T cells and macrophage [51]. Overall, expression of programmed cell death ligand 1 (PD-L1) expression was low in both pituicytomas and SCOs, indicating that classic PD-1/PD-L1 inhibitors alone are unlikely to be effective in these tumors. However, their immune microenvironments diverge significantly. SCOs exhibited a higher proportion CD8 + cytotoxic T lymphocytes (CTLs) and close spatial interactions between macrophages and interferon-gamma (IFN-γ)-producing cells, reflecting an active intrinsic antitumor immune response [51]. In contrast, pituicytomas showed an elevated proportion of exhausted CD8 + T cells characterized by co-expressing multiple inhibitory immune checkpoints (including PD-1, LAG-3, TIM-3, and TIGIT), suggesting an immunosuppressive microenvironment [51].

## Treatment modalities and prognosis

Surgical resection is the main treatment modality [13]. However, in the future, with the advancements in molecular biology and clinical trials, targeted therapies could be incorporated into the treatment algorithm of PPTs.

The standard of care for PPTs is maximum safe surgical resection, typically achieved via a transsphenoidal approach (endoscopic or microscopic), or a transcranial approach for tumors with extensive suprasellar extension [29]. However, gross total resection is challenging. In a recent meta-analysis, complete resection was achieved in only 49% of patients [15]. Subtotal resection is predominantly caused by severe intraoperative bleeding (reported in 30.7% of patients) due to the hypervascular nature of these lesions, especially in SCOs, and firm adherence to the pituitary stalk, optic chiasm, and cavernous sinus [29, 34]. Critical bleeding has been reported in 20% of patients, with a second procedure needed because the first one was prematurely interrupted [15].

Postoperative complications also include hypopituitarism, present in approximately 42% of patients, with involvement of one or more hormonal axes [13]. It is followed by AVP-D (transient or permanent) which occurs in about 33% of patients [15], as well as cerebrospinal fluid leak (2.6%), syndrome of inappropriate antidiuresis (2%), and meningitis (1%) [13]. Close follow-up is essential for early detection and management of these potential complications.

In case of residual mass, tumor remnant can remain stable for a variable period, as described in the literature, consistent with their low-grade behavior, with rare recurrences seen in GCTs [15]. However, a high percentage of recurrence has been documented in up to 44% of pituicytoma [10, 15, 25], and also in up to 66% of SCOs in some cohorts following surgical resection [33, 51]. Ki-67 labeling index is a poor predictor of tumor recurrence [29, 54].

To optimize postoperative surveillance, Das et al. conducted a systematic review to determine clinical and radiological predictors of disease recurrence and persistence [29]. Receiver operating characteristic analysis identified a maximum tumor dimension of 20.5 mm as the optimal threshold for predicting surgical failure and recurrence, with 82% sensitivity and 80% specificity in pituicytomas, and 83% sensitivity and 63% specificity in SCOs. Additional risk factors included male sex for SCO (56% in recurrent vs. 10% in non-recurrent tumors) and headache at presentation for pituicytoma (47% in recurrent vs. 17% in non-recurrent tumors) [29]. Furthermore, follow-up duration was a significant determinant of recurrence detection, highlighting that recurrences can occur late (10 years post-surgery), necessitating lifelong radiological and endocrine surveillance [29].

Recently, a landmark study demonstrated that prognosis varies according to the histological subtype and chromosomal copy number variations (CNVs) [19]. Using DNA methylation array data to infer copy number profiles, Schmid et al. detected no chromosomal changes in 27 of 47 tumors (57%). However, chromosomal imbalances were highly prevalent in SCOs (13 of 21 tumors, 61%), followed by pituicytomas (5 of 14 tumors, 35%), and were rare in GCTs (2 of 12 tumors, 17%) [19]. SCOs displayed a high copy number load, with 47% (10 of 21) showing five or more chromosomal changes [19]. The types of aberrations also differed as shown by another study that analyzed CNV in nine PPTs [49]. Pituicytomas and GCTs exclusively exhibited whole-chromosome or whole-chromosome-arm gains or losses (loss of chromosome 10, 13q, 18q, or 19), whereas SCOs frequently harbored partial-arm intrachromosomal breaks and deletions, such as recurrent loss of chromosome 1p, and homozygous deletions of *CDKN2A/B* and *SMARCB1* – Table 2 [49].

In Schmid et al.’s study, Kaplan-Meier survival analysis demonstrated that the presence of any chromosomal CNV was significantly associated with a shortened progression-free survival across the entire PPT cohort [19]. When stratified specifically within the combined pituicytoma and SCO cohorts, the presence of CNVs remained highly predictive of recurrence. To translate these findings into clinical practice, Schmid et al. proposed a practical histomolecular classification model with 3 subtypes [19]. Subtype 1 consists of pituicytoma or SCO lacking CNV, representing genetically stable tumors that follow a favorable clinical course with low recurrence risk (Table 2).

**Fig. 2:**
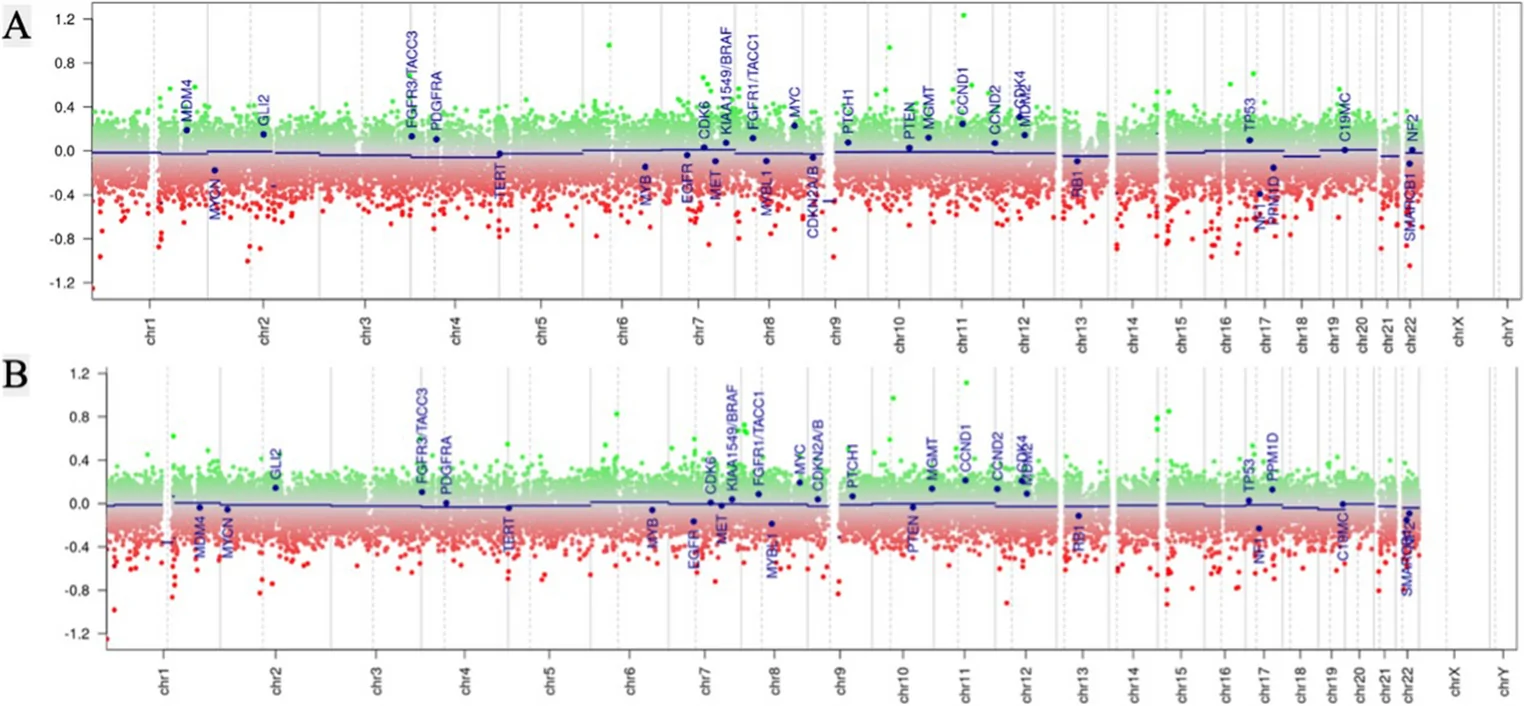
DNA methylation-derived copy number variation plot of a SCO (**A**) and of a pituicytoma (**B**) showing no chromosomal gain or loss

An example is our SCO patient (Fig. 1I-L), which underwent surgery and has a residual tumor that is stable for the last 17 months. No chromosomal gain or loss was seen in the methylation profile – Fig. 2A. Our patient with pituicytoma (Fig. 1A-D) also underwent surgery, but postoperative MRI has not yet been performed. Nevertheless, the likelihood of recurrence is considered low based on absent CNV in the methylation profile – Fig. 2B.

On the other hand, subtype 2 represents pituicytoma or SCOs harboring chromosomal CNVs (genetically unstable tumors), with high rates of recurrence, requiring intensive, long-term radiological surveillance [19]. Subtype 3 consists of GCTs, with or without CNVs, characterized by genomic silence and a favorable clinical course (Table 2).

Regarding radiotherapy, its efficacy, indication or type are not well established in the management of PPTs [15]. While conventional adjuvant radiotherapy (fractionated stereotactic radiotherapy or radiosurgery) is often employed for residual or progressive tumors, its therapeutic efficacy remains controversial, as some patients experience progression despite treatment [50, 51]. However, promising horizons for personalized, targeted therapies are emerging due to novel genomic and immune discoveries.

## Targeted therapies

The presence of targetable *BRAFV600E* variants enables precision treatment with selective BRAF inhibitors (such as dabrafenib or vemurafenib) and MEK inhibitors (such as trametinib), which have achieved durable tumor responses in case reports of refractory SCOs [55, 56]. As pituicytomas are generally positive for VEGF, SST3 and SST5, anti-VEGF therapy (bevacizumab) and somatostatin receptor ligand (pasireotide) could theoretically offer anti-angiogenic and anti-proliferative effects [57].

Pathogenic variants in *SETD2* and *PAX8* have been described in other tumors and could potentially be targetable with existing approved drugs, including immune checkpoint inhibitors and histone deacetylase inhibitors, respectively [53]. However, further research is necessary as to whether tumor growth can be inhibited using these drugs.

Regarding immunotherapeutic strategies, based on Li et al.’s study, the immune exhaustion in pituicytomas provides a biological rationale for exploring alternative immune checkpoint inhibitors, such as LAG-3 or TIM-3 inhibitors [51]. Conversely, in immune-active SCOs, therapeutic strategies designed to enhance CTL activity offer a targeted strategy to control residual or unresectable disease, in theory [51].

No past or ongoing clinical trial was found in the clinicaltrials website for “posterior pituitary tumors”, “pituicytoma”, “spindle cell oncocytoma” or “granular cell tumor”. Therefore, although targeted therapies could be a potential option in patients with refractory PPTs after surgery, further clinical research is necessary [51, 57].

## Ependymal pituicytoma

Ependymal pituicytomas are very rare with fewer than 20 patients published in the literature [8, 12, 13, 58]. In a recent published review including 13 patients, the mean age at presentation was 47 years (range 31–81), with a male predilection [59]. Clinically, patients presented with mass effect and/or symptoms of hypopituitarism. On MRI, the lesion was often solid or cystic-solid, hypointense on T1W and hyperintense on T2W, with well-defined tumor margins upon contrast enhancement.

There is no established treatment, apart from surgical resection. Ependymal pituicytoma displays a spindle cell architecture containing well-formed true ependymal rosettes and perivascular pseudorosettes [13]. Immunohistochemically, GFAP, vimentin, and S100 protein are variably expressed, while EMA shows a strongly positive membranous positivity, with paranuclear dot-like pattern, consistent with ependymal differentiation – Table 1 [58–61]. Staining for CAM 5.2, CD99, Bcl-2, and Galectin-3 can be observed, but inconsistently [58, 62]. Data on molecular features and prognosis are limited due to their rarity.

## Conclusions

PPTs are rare, represent a single histogenetic continuum characterized by TTF-1 expression, and usually present with mass effect and no AVP-D. They should be considered in the differential diagnosis of sellar and suprasellar lesions, as surgical planning is important. The main treatment modality nowadays is surgery, however gross total resection is frequently hindered by hypervascularity and dense tissue adherence. Another treatment option for progressive tumors is radiotherapy. Prognosis is based on chromosomal copy number variation, with GCTs rarely recurring, and pituicytomas or SCOs without CNV showing a more favorable prognosis than those with CNV. Emerging genetic and immune findings could provide a rational for targeted therapy for refractory PPT.

## Data Availability

No datasets were generated or analysed during the current study.
